# The interplay between gastrocnemius medialis force–length and force–velocity potentials, cumulative EMG activity and energy cost at speeds above and below the walk to run transition speed

**DOI:** 10.1113/EP090657

**Published:** 2022-11-17

**Authors:** Andrea Monte, Paolo Tecchio, Francesca Nardello, Beatriz Bachero‐Mena, Luca Paolo Ardigò, Paola Zamparo

**Affiliations:** ^1^ Department of Neurosciences Biomedicine and Movement Sciences University of Verona Verona Italy; ^2^ Human Movement Science Faculty of Sports Science Ruhr University Bochum Bochum Germany; ^3^ Department of Physical Education and Sport Universidad de Sevilla Sevilla Spain; ^4^ Department of Teacher Education NLA University College Oslo Norway

**Keywords:** energy cost, force potentials, locomotion

## Abstract

The aim of this study was to investigate the interplay between the force–length (*F*–*L*) and force–velocity (*F–V*) potentials of gastrocnemius medialis (GM) muscle fascicles, the cumulative muscle activity per distance travelled (CMAPD) of the lower limb muscles (GM, vastus lateralis, biceps femori, tibialis anterior) and net energy cost (*C*_net_) during walking and running at speeds above and below the walk‐to‐run transition speed (walking: 2–8 km h^−1^; running: 6–10 km h^−1^). A strong association was observed between *C*_net_ and CMAPD: both changed significantly with walking speed but were unaffected by speed in running. The *F–L* and *F–V* potentials decreased with speed in both gaits and, at 6–8 km h^−1^, were significantly larger in running. At low to moderate walking speeds (2–6 km h^−1^), the changes in GM force potentials were not associated with substantial changes in CMAPD (and *C*_net_), whereas at walking speeds of 7–8 km h^−1^, even small changes in force potentials were associated with steep increases in CMAPD (and *C*_net_). These data suggest that: (i) the walk to run transition could be explained by an abrupt increase in *C*_net_ driven by an upregulation of the EMG activity (e.g., in CMAPD) at sustained walking speeds (>7 km h^−1^) and (ii) the reduction in the muscle's ability to produce force (e.g., in the *F–L* and *F–V* potentials) contributes to the increase in CMAPD (and *C*_net_). Switching to running allows regaining of high force potentials, thus limiting the increase in CMAPD (and *C*_net_) that would otherwise occur to sustain the increase in locomotion speed.

## Abstract (highlights)

- **What is the central question of the study?**
  Are the changes in force potentials (at the muscle level) related with metabolic changes at speeds above and below the walk‐to‐run transition?
- **What is the main finding and its importance?**
  The force–length and force–velocity potentials of gastrocnemius medialis during human walking decrease as a function of speed; this decrease is associated with an increase in cumulative EMG activity and in the energy cost of locomotion. Switching from fast walking to running is associated to an increase in the force potentials, supporting the idea that the ‘metabolic trigger’ that determines the transition from walking to running is ultimately driven by a reduction of the muscle's contractile capacity.

## 1 INTRODUCTION

Human locomotion is mainly driven by the lower limb muscles, which, during the stance phase of walking and running, produce the force needed to support and accelerate the body within the environment (Cavagna & Kaneko, 1977; Peyré‐Tartaruga et al., 2021). These muscles are almost exclusively responsible for whole‐body energy expenditure (Griffin et al., 2003; Poole et al., 1992). Indeed, it is well recognized that metabolic energy expenditure during locomotion is determined (among others) by the level of muscle activation necessary to generate the force to propel the body forwards (Kipp et al., 2018; Taylor, 1985). This force depends (among others) on the force–length (*F–L*) and force–velocity (*F–V*) potentials of the muscle (Biewener, 1998; Roberts et al., 1997), which are estimates of the fraction of maximum force capability of a muscle according to its *F–L* and *F–V* relationships (Gordon et al., 1966). In other words, the force potentials express the operating length and velocity of the muscle fascicles with respect to their *F–L* and *F–V* relationships.

The operating length or velocity of a muscle (which determines the muscle's potentials) may affect whole‐body metabolic energy expenditure because muscles utilize more ATP per unit of force production when they are activated at shorter lengths than optimal (less economical force production) (Stephenson et al., 1989). Furthermore, for a given level of force, operating at lengths other than optimal and/or at a high contraction velocity requires the body to activate more muscle fibres and/or to increase their firing rate; in both cases this is associated with an increase in metabolic energy expenditure (Beck et al., 2020; Christie et al., 2016). The history dependence of force generation (i.e., increased force after active muscle lengthening (Edman et al., 1982) and decreased force after active shortening (Abbott & Aubert, 1952)) may also influence force production and the cost per unit force (Joumaa & Herzog, 2013); this phenomenon could also explain the lower than expected cost of force production in stretch shortening cycles (Curtin et al., 2019; Holt et al., 2014).

The ankle plantar flexor muscles are a vital source of mechanical power for human locomotion; during walking and running they provide more than 40% of the total mechanical power generated at the whole body level (Arnold et al., 2013; Farris & Sawicki, 2012a). Of this power, about 50% is provided by the contractile (active) component and the other 50% by the elastic components (Farris & Sawicki, 2012b; A. Lai et al., 2014; Monte, Maganaris et al., 2020). The plantar flexor muscles provide bodyweight support, contribute to propulsion and accelerate the limbs into the swing (Sasaki & Neptune, 2006). Moreover, the ankle joint acts in a spring‐like manner (Qiao & Jindrich, 2016), absorbing energy in plantar flexor muscle–tendon units during early stance and providing energy to accelerate the body in late stance (AKM Lai et al., 2019). The contribution provided by the series elastic component of the plantar flexor muscles, estimated using an inverse dynamic approach, remains stable as a function of speed in walking (Farris & Sawicki, 2012b), whereas it increases when running speed increases (Monte, Baltzopoulos et al., 2020).

The force required to produce whole‐body movement during locomotion varies between gaits and across speeds (Arnold et al., 2013; Farris & Sawicki, 2012b; A. Lai et al., 2015); thus, the neuromuscular system may need to adjust the plantar flexors’ *F–L* and *F–V* potentials with gait and speed to meet the energy demands of the muscles.

Due to the steep slope of the hyperbolic *F–V* curve at low to moderate shortening velocities, the *F–V* potential might be particularly sensitive to changes in shortening velocity across speeds. In this regard it was observed that the gastrocnemius medialis (GM) fascicles shorten during the entire stance phase and that their shortening velocity increases as a function of speed (Farris & Sawicki, 2012b; A. Lai et al., 2015; Monte, Baltzopoulos et al., 2020); as a consequence, the GM *F–L* and *F–V* potentials decrease as a function of speed in running (e.g., from 0.92 and 0.94 to 0.83 and 0.85 at speeds of 10 and 16 km h^−1^ (Monte, Baltzopoulos et al., 2020). To our knowledge no in vivo data are reported in the literature regarding the changes in force potentials as a function of speed in walking. Of note, in vitro, changes in the ATPase rate were observed only when fascicle length was <0.75 *L*_0_ (where *L*_0_ is the optimal length) (Stephenson et al., 1989), a value that is hardly reached during human locomotion.

A point of scientific interest when investigating the impact of the changes in *F–L* and *F–V* potentials on the physiological demands of human locomotion, regards the transition between walking and running. Three main theories have been proposed to explain the spontaneous walk to run transition. The first one is based on the idea that gait transition is triggered by metabolic energy expenditure at the whole‐body level; this mechanism was initially proposed by Margaria (1963) based on the observation that humans tend to use speeds closer to the most economical ones at each gait. It was, indeed, observed that the spontaneous transition from walking to running occurs at speeds close to (5–6% slower) those predicted by the metabolic cost hypothesis and that subjects never select to walk/run at speeds close to the speed at which gait transition occurs (Minetti et al., 1994). The second theory is based on the observation that an abrupt increase in muscle activity occurs when walking speed gets close to the transition speed (Abe et al., 2019; Prilutsky & Gregor, 2001); these findings support the idea that gait transition is triggered at the muscle level. A third theory is based on the idea that spontaneous transition speed is determined by the safety maintenance of the musculoskeletal system: it is based on the observation that transition occurs when reaching a given vertical force (peak stress) acting on the muscle–tendon structures (e.g., Farley & Taylor, 1991).

More recently, it was suggested that the transition from walking to running could be triggered by a decrease in the plantar flexor muscle fibres’ ability to produce force (e.g., Farris & Sawicki, 2012b; A. Lai et al., 2015; Minetti et al., 1994; Neptune, 2005). In particular, Neptune and Sasaki (2005) and Farris and Sawicki (2012a) observed that human plantar flexor muscle fibres’ shortening velocity decreases after switching from walking to running at the preferred transition speed. In theory, this improvement in contractile conditions should allow the calf muscles to produce greater forces when running compared with walking at the same speed, allowing them to operate at higher *F–L* and *F–V* potentials, thus, reducing the metabolic energy demands.

Last but not least, a recent study suggested that the transition speed could also be explained by the need to preserve the Achilles tendon mechanical behaviour (i.e., AT force and power) and the ankle spring function (Monte, Tecchio, Nardello & Zamparo, 2022).

From a certain point of view, all these observations could be considered as pieces of the same puzzle: the transition from walking to running could be explained by an abrupt increase in the metabolic energy demands of walking driven by an abrupt upregulation of the EMG activity associated to a reduction in the muscles’ ability to produce force (e.g., in the *F–L* and *F–V* potentials).

Hence, this study aimed to acquire *in viv*o evidence to verify the possible correlations between the changes in *F–L* and *F–V* potentials and the changes in metabolic energy expenditure during walking and running at increasing speeds (above and below the typical transition speed, about 7 km h^−1^). Specifically, we investigated the effects of speed and gait on the *F–L* and *F–V* potentials of the GM to understand whether the transition between gaits could be explained by an increase in the metabolic demand due to an impairment in the muscle's contractile capability (while walking at fast speeds, e.g., >7 km h^−1^).

Based on previous studies (Bohm et al., 2019; Farris & Sawicki, 2012b) we examined three hypotheses: (i) that the GM *F–L* and *F–V* potentials would decrease with walking (and running) speed, impairing the muscle's force capacity; (ii) that the changes in GM *F–L* and *F–V* potentials would correlate with the changes in the energy cost of walking (and running); and (iii) that switching from fast walking to running would slow down fascicle shortening velocity, allowing the *F–L* and *F–V* potentials to increase and metabolic energy expenditure to decrease.

## 2 METHODS

### 2.1 Ethical approval

The local ethical committee approved the experimental protocol (protocol number 2020‐UNVRCLE‐161 0142370) and all subjects gave their written informed consent. The study conformed to the standards set by the version of the *Declaration of Helsinki* that was in place at the time of the experiments.

### 2.2 Participants

Fourteen (seven males and seven females) healthy subjects (age: 27.6 ± 4.6 years; body mass: 63.4 ± 11.6 kg; height: 1.69 ± 0.08 m) participated in this study. All participants were in good health and reported no recent history of lower limb neuro‐musculoskeletal injury.

### 2.3 Experimental design

Each subject participated in two different experimental sessions. In the first session, the *F–L* and *F–V* relationships of GM were determined by means of maximal voluntary contraction tests of the plantar flexors. During these experiments, an ultrasound apparatus was utilized to record the GM muscle fascicle length. The EMG activity of GM and its antagonist muscle (tibialis anterior; TA) was also recorded. In the second session, the kinematics of the body segments, the EMG activity of GM and its muscle fascicle behaviour, were investigated at different speeds (with 1 km h^−1^ increments) during walking (from 2 to 8 km h^−1^) and running (from 6 to 10 km h^−1^) along with metabolic data.

### 2.4 Data collection

#### 2.4.1 Dynamometric measurements

Before the beginning of each test, the participants familiarized with the equipment and procedures. For the plantar flexor measurements, they were secured to a dynamometer (Cybex NORM, Lumex Inc., Ronkonkoma, New York, USA) in a prone lying position with the right knee in the anatomical position and the foot fixed to the dynamometer footplate. Due to the joint rotation that occurs during contractions from rest to maximum effort (Arampatzis et al., 2004), the ankle axis of rotation and the dynamometer axis of rotation were aligned during a preliminary maximal voluntary contraction (MVC). To account for the effects of gravity and passive joint torque on the net joint torque, three passive plantar flexions (15° s^−1^) were performed over the entire range of motion while participants were instructed to relax.

The *F–L* relationship was obtained from MVCs at various joint angles, with the same procedures described in Monte, Baltzopoulos et al. (2020). For the plantar flexors, the right leg was fully extended in the anatomical position and four MVCs were performed from 15° dorsiflexion to 30° plantar flexion (0° is foot at right angles to the shank) at 15° intervals. With this protocol the measured ankle joint moment (at the dynamometer) is the outcome of the action of all the plantar flexor muscles (during in vivo measurements, is not possible to isolate the contribution of GM alone) but, in the fully extended knee joint position, the contribution of GM to the plantar flexor force is the highest (e.g., Rubenson et al., 2012).

During each contraction, the angle and the moment arm of the ankle joint were obtained based on 2D video analysis (Monte, Baltzopoulos et al., 2020). The moment arm was measured as the perpendicular distance from the tendon's line of action to the centre of rotation of the ankle (as proposed by Tecchio et al., 2022) by taking into account the Achilles tendon curvature; the latter was identified by means of six markers: on the tuber calcanei (AT origin), at 2, 4, 6 and 8 cm distance from the tuber calcanei and in the middle of the ultrasound probe (to identify the AT junction with GM). This method allows for a more correct estimation of the Achilles tendon force and power compared to other methods (e.g., straight line method) (Tecchio et al., 2022).

A B‐mode ultrasound scanner (Telemed MicrUs EXT‐1H rev. D, Vilnius, Lithuania) was used to record images with a depth and width of 40 and 60 mm, respectively. To ensure the best image quality, sample frequency was adapted to the individual muscle characteristics (i.e., 90 Hz in eight subjects and 115 Hz in six subjects).

Ultrasound data were recorded from the right GM of each participant. The ultrasound probe was placed in the sagittal plane on the muscle belly at 30% of the distance between the popliteal crease and the malleolus. The position of the scanning probe was corrected until the superficial and deep aponeuroses and the connective tissue that surrounds the muscle fascicles were clearly visible.

The EMG signals of GM, TA, biceps femoris (BF) and vastus lateralis (VL) were collected using a wireless system (Aurion, Cometa, Bareggio (Milano), Italy) sampling at 1000 Hz. Ag–AgCl bipolar electrodes were carefully placed over muscle bellies after the skin surface was prepared by light abrasion and cleaned with an alcohol swab.

Ultrasound and EMG data were recorded synchronously with dynamometer data (angular velocity, moment and position); a digital output generated by the ultrasound scanner triggered all instrumentation.

#### 2.4.2 Walking and running trials

Before the beginning of the test, each subject was familiarized with the equipment and the procedures.

Participants were asked to walk and run on a treadmill (H/P/Cosmos, Saturn 300/100r, Nussdorf‐Traunstein, Germany) with their self‐selected step length and step frequency. Each testing condition (walking at speeds from 2 to 8 km h^−1^ and running at speeds from 6 to 10 km h^−1^) was proposed in a random order and maintained for at least 6 min. Ten minutes of passive recovery were interposed between trials.

A 3D motion capture system (eight cameras; Vicon, Oxford, UK) was used to record the trajectories of 49 markers (a customized full‐body Plugh in Gait), sampling at 200 Hz. The marker set was adapted to measure the Achilles tendon lever arm during locomotion, as described above.

The EMG and the ultrasound data of GM were recorded using the same procedures and instrumentation described for the dynamometric measurements.

During each locomotor trial, the EMG activity of VL, BF and TA was also collected. Ultrasound, kinematic and EMG data were synchronized by a digital output generated by the ultrasound scanner that triggered all instrumentation.

Oxygen uptake (${\dot{V}}_{O_{2}}$) during each walking/running trial was determined by means of a breath by breath metabolimeter (K5, Cosmed, Roma, Italy). Six minutes of baseline values in the standing position were collected before the tests and data were collected for 6 min during exercise; data collected in the last minute of rest/exercise were averaged and used in further analysis.

### 2.5 Data analysis

#### 2.5.1 Assessment of the *F–L* and *F–V* relationship

The total moment generated at the ankle level (measured by means the dynamometer) was corrected for the effect of the gravitational force (Arampatzis et al., 2004; Bakenecker et al., 2019) and for the EMG–moment relationships of the antagonist muscle (Kellis & Baltzopoulos, 1998). The force applied to the Achilles tendon was estimated as the ratio of total ankle joint moment and tendon moment arm, using a 2D approach (Monte, Baltzopoulos et al., 2020).

**FIGURE 1:**
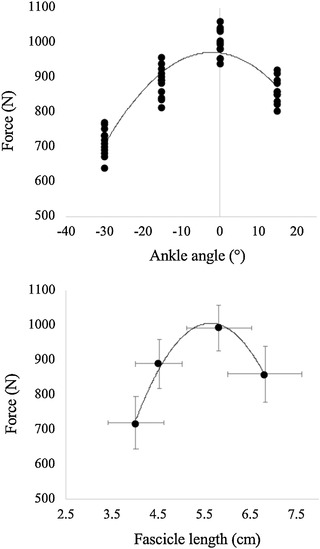
Force–angle (upper panel) and force–length (lower panel) relationships of the plantar flexor muscles as determined during the isometric contractions at the dynamometer. Individual data are reported for the force–angle relationship (*n* = 14 at each angle), while means and standard deviation are reported for the force–length relationship

The *F–L* relationship was determined (for each subject) based on a second‐order polynomial fit by knowing the maximum force obtained during the MVCs and the corresponding muscle fascicle length (Figure 1, lower panel). The maximum force attained at each ankle angle was taken as the maximum possible muscle force at that angle (Figure 1, upper panel). For the ultrasound measurements, fascicle length was manually determined from the ultrasound images using the software ImageJ (NIH, Bethesda, MD, USA); measurements were performed during the isometric phase, once the muscle–tendon unit has reached a steady state. The maximal isometric force applied to the tendon (*F*_max_) and the optimal length (*L*_0_, the length at which this peak occurs) were determined for each subject using his/her own *F–L* curve. The *F–L* potential was calculated based on the normalised fascicle operating range (fascicle length/*L*_0_) throughout the (individual) *F–L* relationships during the stance phase (Bohm et al., 2019, 2021; Monte, Baltzopoulos et al., 2020). The *F–L* potential represents the operating length of the fascicle with respect to the *F–L* curve; in other words, it represents the fraction of the maximum force (*F*/*F*_max_) that a muscle can theoretically reach based on its fascicle behaviour.

The raw EMG signal during the maximal isometric contractions was filtered with a band‐pass third‐order Butterworth filter at 20–450 Hz; the root‐mean‐square (RMS) of the signal was then calculated. The mean RMS value during the steady‐state muscle force was taken as the maximum EMG activity during that contraction. The maximum EMG activity reached during the MVCs was taken as the maximum possible EMG activity (EMG_MAX_) and used for further analysis.

The *F–V* relationship of GM was determined using the classic Hill equation (Hill, 1964) based on the muscle‐specific maximum fascicle shortening velocity (*V*_max_) and the constants *a* and *b*, as previously proposed by Bohm et al. (2019, 2021). Briefly, these calculations derive from the studies of Harber (Harber & Trappe, 2008; Harber et al., 2004), who determined *V*_max_ in vitro for type 1 (0.90 *L*_0_ s^−1^) and type 2 (3.00 *L*_0_ s^−1^) fibres in the human gastrocnemius (in vivo, at physiological temperature, *V*_max_ = 2 and 10 *L*_0_ s^−1^, respectively). Assuming an average fibre type distribution of the human gastrocnemius (50% and 50% for type 1 and 2 fibres) (Edgerton et al., 1975), we calculated a value of *V*_max_ (6.00 *L*_0_ s^−1^) close to that reported by Monte, Baltzopoulos et al. (2020) during in vivo evaluations (e.g., 5.97 *L*_0_ s^−1^, in a group of young and healthy adults). Coefficient *a* was then calculated as 0.1 + 0.4FT, where FT is the fast twitch fibre type fraction (0.5, see above); the product of *a* and *V*_max_ then gives coefficient *b* (see also Bohm et al., 2019, 2021).

The *F–V* potential was calculated based on the normalised fascicle velocity (fascicle velocity/*V*_max_) throughout the (individual) *F–V* relationships during the entire stance phase (Bohm et al., 2019, 2021; Monte, Baltzopoulos et al., 2020). The *F–V* potential represents the velocity of the fascicle with respect to the *F–V* curve; in other words, it represents the fraction of the maximum force (*F*/*F*_max_) that a muscle can theoretically reach based on its fascicle contraction velocity.

#### 2.5.2 Walking and running measurements

Kinematic and ultrasound data were analysed for 10 stance phases in the last minute of exercise during both walking and running; for each, instrumentation data were interpolated to 200 sample points. The walk‐to‐run transition speed was estimated for each subject as: *v* = $\sqrt{0.5\mathrm{gL}}$; where 0.5 is the Froude number that corresponds to the transition speed, *g* is the gravitational acceleration and *L* the leg length (Alexander, 1989).

Marker trajectories were filtered with a forward and reverse second order, low pass Butterworth filter, with a cut‐off frequency of 12 Hz. Inverse kinematics was used to calculate the angular rotation for each body segment. Data were analysed with a custom‐written software (LabVIEW 10, National Instruments, Austin, TX, USA).

The EMG activity of VL, GM, TA and BF was analysed for 10 stride phases. The raw EMG signal collected during walking and running was filtered with a band‐pass third‐order Butterworth filter at 20–450 Hz and the RMS was calculated using a moving window of 25 ms throughout the entire stride. Then, the RMS was normalized for the maximum EMG activity assessed during the dynamometric trials (RMS_N_) (Pincheira et al., 2017). The cumulative activity per distance travelled (CMAPD) was calculated for each muscle as proposed by Carrier et al. (2011) as the ratio of RMS_N_ and walking/running speed (e.g., CMAPD_VL_, CMAPD_GM,_ CMAPD_TA,_ CMAPD_BF_). Total CAMPD (CMAPD_TOT_) was calculated as the sum of the CMAPD of all the investigate muscles (e.g., Pincheira et al., 2017). Even though CMAPD_TOT_ is only a rough estimation of the active muscle volume, the integration of the EMG signals from different muscles provides a reliable correlative indication of muscle metabolism (Blake & Wakeling, 2013; Carrier et al., 2011).

For the ultrasound measurements fascicle length data were post‐processed from the images using a customized version of an automatic tracking algorithm (e.g., van der Zee & Kuo, 2021). At the end of the auto‐tracking, every frame of the tracked fascicle lengths was visually examined to check the algorithm accuracy (approximately 40% of the tracked fascicles have been manually corrected). Whenever the fascicle length was deemed inaccurate, the two points defining the muscle fascicles were manually repositioned.

**FIGURE 2:**
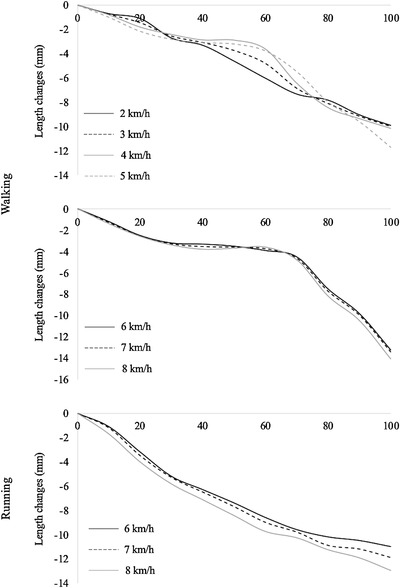
Fascicle length behaviour of gastrocnemius medialis during the stance phase of walking (upper and middle panels) and running (lower panel) at all the investigated speeds. Data refer to mean values among subjects (*n* = 14 at each speed). Length values are ‘normalized’ for the initial length at touch‐down

The fascicle length behaviours of GM during the stance phase of walking and running at all the investigated speeds are reported in Figure 2. In this figure, length values are ‘normalized’ for the initial length at touch‐down (i.e., data represent length changes).

Muscle fascicle velocity was calculated as the first derivative of its length changes. Fascicle length and fascicle velocity were normalized to the optimal fascicle length (*L*/*L*_0_) and maximum shortening velocity (*V*/*V*_max_), respectively (as determined during the dynamometric measurements). The *F–L* and the *F–V* potentials of the GM muscle fascicles were calculated based on data of fascicle behaviour and shortening velocity, as proposed in other studies.

Oxygen uptake (${\dot{V}}_{O_{2}}$) at rest was subtracted from ${\dot{V}}_{O_{2}}$ during exercise, in order to obtain net oxygen uptake (${\dot{V}}_{O_{2}\mathrm{net}}$, expressed in mlO_2_ min^−1^ kg^−1^). Net energy cost (*C*_net_) was calculated as: ${\dot{V}}_{O_{2}\mathrm{net}}$/*v* (where *v* is the treadmill speed) and expressed in J m^−1^ kg^−1^ by using an energy equivalent (EQ, J ml O_2_^−1^) that takes into account the respiratory exchange ratio (RER): EQ = 4.94, RER = 16.04.

### 2.6 Statistical analysis

Values are presented as means ± SD. A two‐way repeated‐measures ANOVA (with main factors: speed and gait) with a Bonferroni adjustment was used to test for differences across speeds and between gaits in all the investigated variables. The correlations between *C*_net_, CMAPD_TOT_ and the *F–L* and *F–V* potentials were assessed using Pearson's correlation coefficient. However, due to the fact that false positive correlations increase with the number of correlations performed, Pearson's product moment correlation *P*‐values were corrected for multiple tests using the Benjamini–Hochberg procedure (Benjamini & Hochberg, 1995) with a false detection rate of 5% (significance was defined as adjusted *P* < 0.05). Statistical analyses were performed using SPSS Statistics (Version 20.0, IBM Corp., Armonk, NY, USA) and the level of significance was set to α = 0.05.

## 3 RESULTS

Significant main effects of speed and gait (and significant interactions) were observed for all the investigated parameters. Since we were not interested in the effect of gait per se in the following sections, the *P*‐values refer to the main effect of speed and to the comparison between walking and running at paired speeds.

**FIGURE 3:**
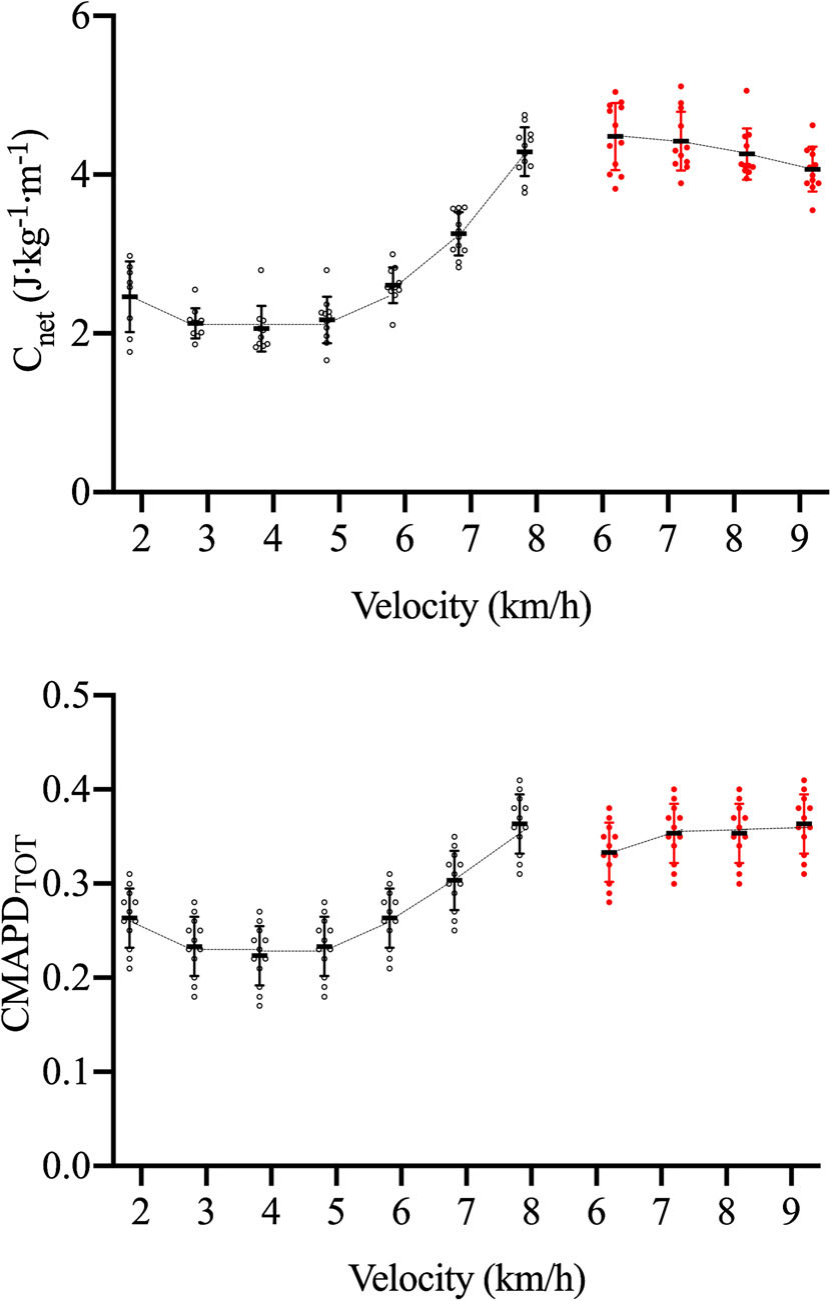
Net energy cost (*C*_net_, upper panel) and total cumulative EMG activity per distance travelled (CMAPD_TOT_, lower panel) as a function of walking (black dots) and running (red dots) speed. Each dot refers to a single participant (*n* = 14 at each speed)

The estimated walk‐to‐run transition speed was 7.5 ± 0.1 km h^−1^. Net energy cost (*C*_net_) changed significantly (*P* = 0.0002) with walking speed, following the typical U‐shape behaviour; in running *C*_net_ was unaffected by speed (*P* = 0.261) (see Figure 3, upper panel). *C*_net_ was larger in running than in walking at 6 and 7 km h^−1^ (*P* = 0.032 in both cases), while no significant difference was observed at 8 km h^−1^.

CMAPD_TOT_ and CMAPD_GM_ changed significantly (*P* = 0.00084) with walking speed but were unaffected by speed in running (see Figure 3 lower panel). As reported for *C*_net_, significant differences were observed in CMAPD_TOT_ and CMAPD_GM_ between walking and running at 6 and 7 km h^−1^, but not at 8 km h^−1^.

**FIGURE 4:**
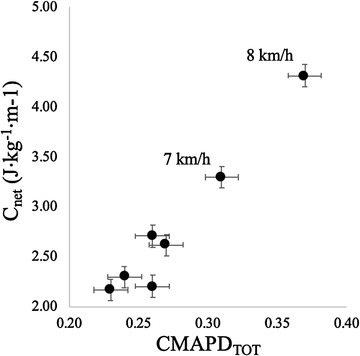
Relationship between CMAPD_TOT_ and *C*_net_ at the investigated walking speeds. An increase in CMAPD_TOT_ above 0.30 is associated with a steep increase in *C*_net_ (at speeds of 7–8 km h^−1^). Data are means and standard deviation (*n* = 14 at each speed)

The mean values of *C*_net_ are reported as a function of the mean values of CMAPD_TOT_ in Figure 4: an increase in CMAPD_TOT_ above 0.30 was associated with a steep increase in *C*_net_ (at speeds of 7–8 km h^−1^).

Data of fascicle length, maximum fascicle shortening and fascicle contraction velocity of GM are reported in Table 1: significant changes were observed as a function of speed in both gaits (*P* = 0.002 and *P* = 0.033 for walking and running, respectively). At matched speeds (6, 7 and 8 km h^−1^), fascicle length changes and maximum fascicle shortening were larger in walking than in running (*P* = 0.0074). GM fascicle contraction speed showed significant differences between walking and running only at 7 and 8 km h^−1^ (see Table 1).

**TABLE 1:** Average values (±SD) of fascicle length, maximum fascicle shortening and fascicle velocity during the stance phase in all conditions (speed and task); the F‐L and F‐V potentials are also reported in the last two columns (*n* = 14 at each speed). Significant differences between walking and running at paired speeds (paired Student's test, *P* values) are reported in parentheses

	Fascicle length (cm)	Max fascicle shortening (cm)	Fascicle velocity (cm s^−1^)	*F–L* potential	*F–V* potential
Walking					
2 km h^−1^	−4.76 ± 0.31	−9.87 ± 0.38	3.70 ± 0.30	0.90 ± 0.02	0.58 ± 0.03
3 km h^−1^	−4.59 ± 0.32	−9.98 ± 0.39	3.33 ± 0.33	0.93 ± 0.03	0.60 ± 0.03
4 km h^−1^	−4.41 ± 0.32	−10.1 ± 0.43	3.44 ± 0.34	0.95 ± 0.03	0.63 ± 0.02
5 km h^−1^	−4.59 ± 0.31	−11.65 ± 0.45	3.44 ± 0.33	0.94 ± 0.02	0.62 ± 0.03
6 km h^−1^	−4.78 ± 0.29 ^(0.034)^	−13.2 ± 0.47 ^(0.004)^	3.56 ± 0.32	0.90 ± 0.03 ^(0.025)^	0.58 ± 0.03
7 km h^−1^	−4.9 ± 0.28 ^(0.0002)^	−13.45 ± 0.51 ^(0.002)^	3.63 ± 0.34 ^(0.034)^	0.86 ± 0.03 ^(0.002)^	0.55 ± 0.03 ^(0.021)^
8 km h^−1^	−5.1 ± 0.30 ^(0.0001)^	−14.01 ± 0.53 ^(0.014)^	3.98 ± 0.33 ^(0.007)^	0.83 ± 0.02 ^(0.0005)^	0.54 ± 0.03 ^(0.0007)^
Running					
6 km h^−1^	−6.5 ± 0.36	−11.00 ± 0.36	3.50 ± 0.28	0.96 ± 0.02	0.60 ± 0.03
7 km h^−1^	−6.99 ± 0.33	−11.90 ± 0.36	3.25 ± 0.23	0.95 ± 0.03	0.61 ± 0.03
8 km h^−1^	−7.54 ± 0.34	−12.9 ± 0.40	3.00 ± 0.21	0.93 ± 0.03	0.68 ± 0.04
9 km h^−1^	−7.66 ± 0.36	−13.1 ± 0.39	3.00 ± 0.23	0.91 ± 0.03	0.65 ± 0.04
10 km h^−1^	−7.99 ± 0.30	−13.06 ± 0.38	2.75 ± 0.22	0.90 ± 0.03	0.54 ± 0.05

### 3.1 GM *F–L* and *F–V* potentials

Maximum isometric force of the plantar flexor muscles, optimal fascicle length and maximum shortening velocity of the GM fascicles, as determined based on the *F–L* and *F–V* relationships were 1002 ± 75 N, 5.8 ± 0.6 cm and 34.8 ± 3.7 cm⋅s^−1^, respectively.

**FIGURE 5:**
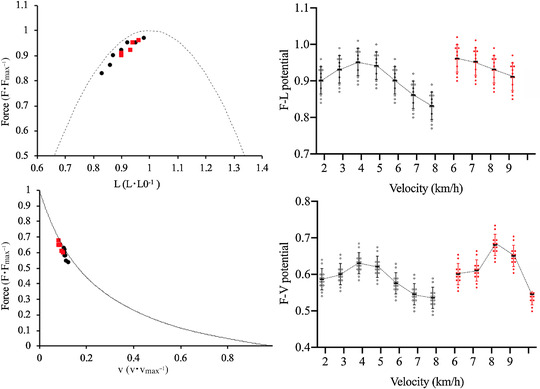
Gastrocnemius medialis *F–L* (upper panels) and *F–V* (lower panels) potentials during walking (black dots) and running (red dots) at the investigated speeds (each dot represents the mean value of 14 subjects). Panels on the left: force potentials are shown with the normalised *F–L* and *F–V* relationships; force is normalized to the maximum force (*F*_0_, determined during maximal isometric plantar flexion contractions), fascicle length is normalized to the optimal fascicle length (*L*_0_, determined during maximal isometric plantar flexion contractions) and fascicle velocity is normalized to the maximum shortening velocity (*V*_max_). Panels on the right: *F–L* and *F–V* potentials as a function of speed during walking (black dots) and running (red dots) (*n* = 14 at each speed); at 8 km h^−1^, shifting from walking to running allows for an increase in the *F–L* and *F–V* potentials of 10% and 20%, respectively

The GM *F–L* and *F–V* potentials (during the stance phase of walking and running) are reported in Figure 5 (and Table 1); force potentials were significantly affected by speed in both tasks (*P* < 0.0001). During walking, the *F–L* and the *F–V* potentials reached their maximum value at speeds of 3–5 km h^−1^. During running, the *F–L* potential steadily decreased as a function of speed, whereas the *F–V* potential showed a maximum around 8 km h^−1^. At matched speeds (6, 7 and 8 km h^−1^), the *F–L* potentials were higher for running than walking while the *F–V* potentials showed significant differences between tasks only at 7 and 8 km h^−1^. At 8 km h^−1^ the *F–L* and the *F–V* potentials were 10% and 20% larger in running than in walking.

The GM force potentials were strongly correlated with CMAPD_GM_ at all speeds and for both tasks: the lower the force potentials the higher the GM EMG activity. Correlation coefficients ranged from −0.75 (*P* = 0.0043) to −0.80 (*P* = 0.0036) for the *F–V* potential and from −0.70 (*P* = 0.0067) to −0.77 (*P* = 0.0040) for the *F–L* potential. The GM force potentials were correlated also with CMAPD_TOT_ (at all speeds and for both tasks): correlation coefficients ranged from −0.60 (*P* = 0.036) to −0.64 (*P* = 0.012) for the *F–V* potential and from −0.58 (*P* = 0.033) to −0.63 (*P* = 0.014) for the *F–L* potential.

**FIGURE 6:**
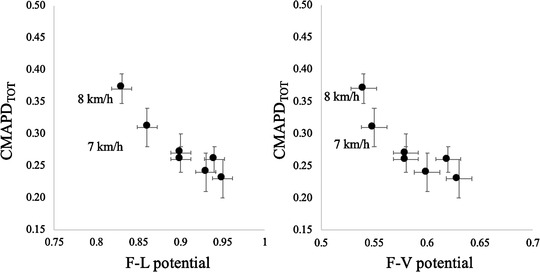
Relationships between total cumulative activity per distance travelled (CMAPD_TOT_) and GM *F–L* (panel on the left) and *F–V* (panel on the right) potentials at the investigated walking speeds (each dot represents the mean value of 14 subjects). Reductions in *F–L* and *F–V* potentials below 0.9 and 0.58, respectively, are associated to a steep increase in CMAPD_TOT_. Data are means and standard deviation. For the *F–L* and *F–V* potentials of GM during running at the other speeds see Table 1

In Figure 6, the mean values of the GM *F–L* and *F–V* potentials are reported as a function of the mean values of CMAPD_TOT_; the negative association between force potentials and CMAPD_TOT_ is particularly evident at speeds of 7–8 km h^−1^ (e.g., when the *F–V* potential was <0.90 and the *F–V* potential was <0.58).

The associations between *C*_net_ and GM *F–L* and *F–V* potentials, as well as between *C*_net_ and both CMAPD_TOT_ and CMAPD_GM_, were also investigated within each walking/running speed; the Pearson's product moment correlation coefficients (*r*) of these relationships are reported in Table 2. At any given speed, and for both tasks, the cumulative activity per distance travelled was positively correlated with *C*_net_: the higher CMAPD the larger *C*_net_. The stronger association was observed with CMAPD_TOT_ but *C*_net_ was significantly correlated with CMAPD_GM_ too.

**TABLE 2:** Pearson's product moment correlation coefficient (*r*) between the energy cost of locomotion (*C*_net_) and the GM *F–L* and *F–V* potentials in walking and running at different speeds (*n* = 14 at each speed) and between *C*_net_ and CMAPD_GM_ and CMAPD_TOT_. Significant differences between walking and running at paired speeds (paired Student's test, *P* value) are reported in parentheses

	GM *F–L* potential	GM *F–V* potential	CMAPD_GM_	CMAPD_TOT_
Walking				
2 km h^−1^	−0.54 ^(0.041)^	−0.62 ^(0.017)^	0.53 ^(0.042)^	0.67 ^(0.009)^
3 km h^−1^	−0.56 ^(0.038)^	−0.64 ^(0.012)^	0.54 ^(0.041)^	0.67 ^(0.009)^
4 km h^−1^	−0.55 ^(0.040)^	−0.59 ^(0.030)^	0.55 ^(0.040)^	0.69 ^(0.008)^
5 km h^−1^	−0.57 ^(0.032)^	−0.60 ^(0.023)^	0.52 ^(0.042)^	0.70 ^(0.006)^
6 km h^−1^	−0.56 ^(0.038)^	−0.63 ^(0.014)^	0.53 ^(0.042)^	0.73 ^(0.004)^
7 km h^−1^	−0.55 ^(0.040)^	−0.64 ^(0.012)^	0.54 ^(0.041)^	0.68 ^(0.008)^
8 km h^−1^	−0.54 ^(0.041)^	−0.63 ^(0.014)^	0.54 ^(0.041)^	0.72 ^(0.006)^
Running				
6 km h^−1^	−0.55 ^(0.040)^	−0.61 ^(0.020)^	0.52 ^(0.042)^	0.68 ^(0.009)^
7 km h^−1^	−0.55 ^(0.040)^	−0.62 ^(0.017)^	0.51 ^(0.043)^	0.68 ^(0.009)^
8 km h^−1^	−0.57 ^(0.032)^	−0.60 ^(0.023)^	0.50 ^(0.044)^	0.67 ^(0.009)^
9 km h^−1^	−0.56 ^(0.038)^	−0.62 ^(0.017)^	0.52 ^(0.042)^	0.69 ^(0.008)^
10 km h^−1^	−0.56 ^(0.038)^	−0.60 ^(0.023)^	0.53 ^(0.042)^	0.68 ^(0.009)^

At any given speed, and for both tasks, the GM *F–L* and *F–V* potentials were negatively correlated with *C*_net_: the higher the force potentials the lower *C*_net_.

## 4 DISCUSSION

In this study, we provided evidence that the GM *F–L* and *F–V* potentials are affected by walking and running speed: the GM muscle's contractile capability decreases as a function of speed in both tasks. Moreover, we observed that changes in GM muscle's potentials at sustained walking speeds (7–8 km h^−1^) are associated with substantial changes in CMAPD_TOT_ which, in turn, are associated with a steep increase in the energy cost of walking. This suggests that a reduction in the GM force potentials leads to an increase in the EMG activity required to sustain contraction, thereby increasing the energy demands of walking. This line of reasoning is reinforced by the correlations among parameters that indicate a between‐subject effect at any given speed.

Based on these results, it can be speculated that the walk‐to‐run transition could be triggered by (among others) the need to preserve the force potentials above a certain value (0.9 and 0.58 for the *F–L* and *F–V* potential, respectively) in the attempt to reduce the increase in the EMG activity needed to sustain contraction, and the associated increase in energy cost, which would otherwise occur when locomotion speed increases.

### 4.1 The GM *F–L* and *F–V* potentials during walking and running

In this study we investigated GM behaviour because the plantar flexor muscles play a pivotal role in human locomotion (Arnold et al., 2013; AKM Lai et al., 2019) and because we previously observed significant relationships between GM fascicle behaviour and whole body energy expenditure during walking (Monte, Tecchio, Nardello, Bachero‐Mena et al., 2022) and running (Monte, Maganaris et al., 2020).

In a modelling study, Neptune and Sasaki (2005) suggested that the inability of the plantar flexor muscles to provide large forces during the propulsive phase of walking might be a determinant of the preferred walk‐to‐run transition speed. These authors observed that switching to running at the preferred speed improves muscle contractile capability by reducing GM muscle contraction velocity and by shifting the fibres’ length operating range closer to the optimal length. In a more recent study (Farris & Sawicki, 2012b) it was observed that the shortening velocity of GM fascicles increases with walking speed and that this impairs the muscle's ability to produce force. These authors further observed that switching to a running gait allows for a reduction in fascicle velocity, and that this was associated with an increase in peak and average muscle force production. Our data confirm and extend their results. Indeed, we observed an increase in the *F–L* and *F–V* potentials at speeds larger than the walk to run transition, and this indicates that the GM muscle fascicles operated closer to their optimal length, and at lower contractions speed, during running (compared to walking).

The behaviour of the GM *F–L* and *F–V* potentials as a function of speed reported in this study is of particular interest for understanding not only the possible determinants of the walk‐to‐run transition but also other features of human locomotion. Indeed, within the range of the investigated walking speeds, both force potentials showed a parabolic trend with a maximum at a speed of about 4 km h^−1^ (e.g., the speed at which *C*_net_ is minimized); walking at speeds higher or lower than 4 km h^−1^ is associated to lower GM *F–L* and *F–V* potentials. Thus, taking into consideration that the pendulum minimizing mechanism is the main determinant of optimal speed in walking, the present study indicates that factors linked to transmission (machine) efficiency and muscle (motor) efficiency help in explaining this fundamental issue of terrestrial locomotion.

Switching from fast walking to running allows for an improvement in the *F–L* and *F–V* potentials. At paired speeds (6–8 km h^−1^) the *F–L* potential is higher in running than in walking, but the difference increases as a function of speed (e.g., from this point of view, switching from walking to running is less ‘convenient’ at 6 than 8 km h^−1^). On the other hand, the *F–V* potential is higher in running than in walking at 7–8 km h^−1^ with no significant differences at 6 km h^−1^, and this indicates that the transition is more ‘convenient’ at 7–8 km h^−1^. Therefore, the operating length and velocity of the GM muscle fascicles could, at least partially, explain the walk‐to‐run transition through their effect on the *F–L* and *F–V* potentials.

### 4.2 The interplay between energy cost, CMAPD and the *F–L* and *F–V* potentials

The observation that a decrease in the muscles’ force contractile capacity is associated to an increase in the metabolic energy required to sustain contraction and, therefore, in the energy cost of locomotion is supported by animal studies (Biewener, 1998; Roberts & Azizi, 2011; Roberts et al., 1997), as well as by recent in vivo human studies (running at 10 km h^−1^; Bohm et al., 2019).

Previous studies have shown that the correlation between *C*_net_ and force potentials can be explained by the EMG activity of the recruited muscles. Indeed, a decrease in the muscle force potentials requires an upregulation of muscle activation to maintain the same level of force (Beck et al., 2020; Roberts et al., 1997). Therefore, when the force potentials decrease, higher levels of EMG activity are necessary to sustain contraction, thereby increasing the energy cost of locomotion. Our data support this notion; indeed, we observed a positive correlation between *C*_net_ and CMAPD_TOT_ as a function of walking speed as well as a negative correlation between CMAPD_TOT_ and the force potentials. These results indicate that when the walking speed increases, the GM force potentials decrease, requiring more EMG activity to sustain the mechanical demands, and this ultimately leads to an increase in the energy expenditure. Of note, we also observed these correlations within each walking speed too (between‐subject effect).

In the case of running, we observed a decrease in the *F–L* and *F–V* potential at increasing speed (within‐subject effect) but these changes were not accompanied by changes in energy cost or CMAPD_TOT_. However, in agreement with the literature (Bohm et al., 2019), we observed significant negative correlations between force potentials and *C*_net_ and positive correlations between CAMPD_TOT_, and *C*_net_, at each running speed (between‐subject effect).

These differences between gaits can be explained by the role of the plantar flexors’ elastic elements. The contribution of the Achilles tendon to the total mechanical power generated by the ankle, as well as the ankle spring‐like function, starts to decrease at walking speeds >6 km h^−1^ (Monte, Tecchio, Nardello, Bachero‐Mena et al., 2022), whereas they steadily increases when running speed increases (A. Lai et al., 2014; Monte, Baltzopoulos et al., 2020; Monte, Maganaris et al., 2020); this possibly counteracts the increases in *C*_net_ imposed by the changes in the force potentials (Monte, Maganaris et al., 2020). The predominant role of the elastic energy recoil could, then, offset the influence of the GM *F–L* and *F–V* potentials in determining CMAPD_TOT_ and hence *C*_net_ at increasing running speeds.

A key finding of our study is represented by the shape of the relationship between CMAPD_TOT_, *C*_net_ and the force potentials in walking, which show a steep increase at speeds of 7–8 km h^−1^. From 2 to 6 km h^−1^ the changes in GM force potentials are not accompanied by substantial changes in CMAPD_TOT_ and *C*_net_. A mechanism that could potentially explain this phenomenon regards the energy recovery, a parameter that quantifies the ability to save mechanical energy by using a pendulum‐like motion (Cavagna & Legramandi, 2020; Monte, Tecchio, Nardello, Bachero‐Mena et al., 2022). Energy recovery shows an opposite trend compared to CMAPD_TOT_ or *C*_net_ (i.e., reaching a maximum where the energy cost is minimized; Cavagna & Kaneko, 1977; Saibene & Minetti, 2003). It is thus possible to speculate that, during walking at 2–6 km h^−1^, the negative effect of a decrease in GM force potentials is counteracted by an increase in energy recovery, minimizing the increase in metabolic energy required to sustain contraction that would otherwise occur.

At walking speeds of 7–8 km h^−1^, the steep increase in *C*_net_ and CMAPD_TOT_ when the force potentials decrease above a certain value can be explained by considering that the oxygen requirement of a muscle is strongly associated with the number of neural pulses provided to the muscle (Fales et al., 1960). This is reflected in the strong relationship between oxygen consumption and the integrated EMG amplitude in human muscles (Bigland‐Ritchie & Woods, 1976). A muscle needs to produce larger forces at faster shortening speeds to drive motion, and the mechanical disadvantage imposed by the decrease in *F–L* and *F–V* potential at sustained walking speeds possibly exacerbates this effect. As recently shown by Monte, Tecchio, Nardello & Zamparo (2022), the elastic energy provided by the Achilles tendon (estimated by means of an inverse dynamic approach) is reduced at high walking speed (>6 km h^−1^); thus, the contribution of the Achilles tendon in determining the mechanical power generated by the ankle decreases and this is associated with an increase in the contribution of the contractile components of the plantar flexors’ muscle–tendon unit (Monte, Tecchio, Nardello & Zamparo, 2022).

Thus, combining our results with those recently published by Monte, Tecchio, Nardello and Zamparo (2022), it seems plausible that the transition from walking to running (which typically occurs between 7 and 8 km h^−1^) could be explained by a combination of mechanical factors that involve, among the others, a decrease in the force potentials and a reduction of the ankle spring‐like function. These mechanical alterations ultimately lead to an increase in metabolic energy expenditure; to avoid it, people switch from walking to running (regaining high force potentials and improving the ankle spring‐like function).

### 4.3 Methodological considerations

Despite our analysing the influence of the GM *F–L* and *F–V* potentials in determining the energy cost during walking and running at speeds close to the transition, we did not determine the actual walk‐to‐run transition speed in each subject. Therefore, we cannot map individual muscle fascicle changes to the corresponding walk to run transition. This can be considered a limitation of the study.

Other propulsive muscles, with larger physiological cross sectional and volume (e.g., soleus, VL and BF) are expected to play a significant role in determining metabolic energy expenditure in human locomotion. The relationships between *C*_net_ and CMAPD is indeed stronger when other muscles than GM alone are taken into consideration and it is reasonable to assume that the changes in EMG activity in these muscles are associated with changes in their force potentials, as observed here for the GM. Future studies on other lower limb muscles would shed further light on the determinants of the cost of transport in human locomotion.

The *F–V* curve can be obtained in vivo by using iso‐velocity contractions but for the plantar flexor muscles these measurements require the determination of specific control variables (e.g., 3D foot kinematics) not available in the lab where the Cybex measurements were performed. For this reason, we based our calculations on recommendations for modelling approaches, as previously suggested in the literature. Since the *F–V* curve and the *F–V* potential can be affected by ‘incorrect estimations’ of *V*_max_, as suggested by Bohm et al. (2019), we conducted a sensitivity analysis by substantially reducing or increasing *V*_max_ by 25%. The GM *F–V* potential changed by only 4%, without significant changes in its behaviour as a function of walking and running speed. These results, which are similar to those reported by Bohm et al. (2019), thus support the robustness of our primary outcomes. To note, the values of *V*_max_ estimated in this study are close to those reported in young and healthy adults (e.g., Hauraix et al., 2015).

The *F–L* relationship was determined by changing the ankle angle while maintaining the knee fully extended; in these conditions all plantar flexor muscles contribute to the ankle joint moment, but the GM mechanical contribution is the highest (Rubenson et al., 2012). Even if this is a limitation of the study, during in vivo measurements it is not possible to isolate the contribution of GM to the moment/force output and our work shares this limitation with other papers published in the literature on this topic.

Last but not least, as shown by Arampatzis et al. (2006), the optimal length of GM (during MVC, as in our case) is not affected by knee angle (even if the GM is a biarticular muscle) at least in the range of knee and joint angles investigated in this study (and typical of human locomotion). Thus, we are confident that the estimates of optimal fascicle length (necessary to calculate the *F–L* potential) were not affected by this issue.

## 5 CONCLUSION

In this study we provided evidence that the *F–L* and *F–V* potentials of GM during human walking and running decrease as a function of speed and are related with the EMG activity of GM (CMAPD_GM_), with the cumulative activity of the lower limb muscles (CMAPD_TOT_) and with the energy cost of locomotion: the lower the force potentials, the larger CMAPD and *C*_net_. Switching to running at speeds of 7–8 km h^−1^ allows the regaining of high force potentials, thus limiting the increase in CMAPD (and *C*_net_) that would otherwise occur when locomotion speed increases. Therefore, the mechanisms at the basis of the walk to run transition could be, at least partially, attributed to a reduction of the GM *F–L* and *F–V* potentials at fast walking speeds, which induces a steep increase in the energy cost of walking.

## AUTHOR CONTRIBUTIONS

Andrea Monte, Francesca Nardello, Paolo Tecchio and Paola Zamparo conceptualized the work and design. Andrea Monte, Paolo Tecchio, Francesca Nardello and Beatriz Bachero‐Mena contributed to data collection and analysis. Andrea Monte, Francesca Nardello and Paolo Tecchio were responsible for statistical analysis and data organization with support from Paola Zamparo. Andrea Monte and Paola Zamparo wrote the manuscript. All authors edited the work. All authors have read and approved the final version of this manuscript and agree to be accountable for all aspects of the work in ensuring that questions related to the accuracy or integrity of any part of the work are appropriately investigated and resolved. All persons designated as authors qualify for authorship, and all those who qualify for authorship are listed.

## CONFLICT OF INTEREST

None.

## FUNDING

None.

## Supporting information

Statistical Summary Document

## Data Availability

These data have not been made publicly available. The corresponding author can provide further information of the data upon reasonable request.
