# Supplementary material for: The interplay between gastrocnemius medialis force–length and force–velocity potentials, cumulative EMG activity and energy cost at speeds above and below the walk to run transition speed
Source: Exp Physiol. 2022 Nov 17;108(1):90–102. doi: 10.1113/EP090657 (PMC10103772; doi:10.1113/EP090657)
Supplement: Supplementary file 1 — Statistical Summary Document [file EPH-108-90-s001.docx]

**Manuscript Title: The interplay between gastrocnemius medialis F-L and F-V potentials, active muscle volume and energy cost at speeds above and below the walk to run transition speed**

**Authors:** Andrea Monte, Paolo Tecchio, Francesca Nardello, Beatriz Bachero-Mena, Luca Paolo Ardigò, Paola Zamparo

**Underlying hypothesis:** we examined three hypotheses: i) the GM F-L and F-V potentials would decrease with walking (and running) speed, impairing the muscle’s force capacity; ii) the changes in GM F-L and F-V potentials would correlate with the changes in the energy cost of walking (and running); iii) switching from fast walking to running would slow down fascicle shortening velocity, allowing the F-L and F-V potentials to increase and metabolic energy expenditure to decrease.

**Definitions of ‘n’:**

n = number of participants

**Statistical summary table:**

| Experimental question number* | Finding/ conclusion | Experimental location/ variable | Mean value | SD | n | Exact P value | Units | Data comparisons | Statistical test | Any other variable | Figure/ table in which data are presented | Comments  e.g. observation |
| --- | --- | --- | --- | --- | --- | --- | --- | --- | --- | --- | --- | --- |
| 1. | F-L and F-V potentials were affected by walking and running speed | F-L vs. speed  F-V vs. speed | F-L potential during walking  From 0.90  to  0.83  F-V potential during walking  From 0.58  to 0.54  F-L potential during running  From 0.96  to  0.90  F-V potential during running  From 0.60  to  0.54 | F-L potential during walking  From 0.02  to  0.02  F-V potential during walking  From 0.03  to 0.03  F-L potential during running  From 0.02  to  0.03  F-V potential during running  From 0.03  to  0.05 | 14 | Main effect of speed on F-L and F-V potentials = P<0.0001 | None | F-L vs. speed  F-V vs. speed | two-way repeated-measures ANOVA with a Bonferroni adjustment | None | Figure 5 and Table 1 | F-L and F-V potentials were affected by walking and running speed |
| 2 | The energy cost (Cnet) of walking was affected by speed whereas this was not the case for running | Cnet vs. walking speed  Cnet vs. running speed | Cnet during walking  From 2  to  4.5  Cnet during running  From 4.3  to  3.9 | Cnet during walking  From 0.3  to  0.6  Cnet during running  From 0.3  to  0.4 | 14 | Main effect of speed on Cnet: P<0.0001 and P=0.872 for walking and running | J/kg/m | Cnet vs. walking speed  Cnet vs. running speed | two-way repeated-measures ANOVA with a Bonferroni adjustment | None | Figure 3 | The energy cost (Cnet) of walking was affected by speed whereas this was not the case for running |
| 3 | At any given speed, and for both tasks, the F-L and F-V potentials were negatively correlated with Cnet: the higher the force potentials the lower Cnet. | Cnet vs. F-L potential  Cnet vs. F-V potential | Correlations coefficients between Cnet vs. F-L in walking and running ranged from: -0.54 and -0.57 for walking  and  -0.55 and -0.57 for running  Correlations coefficients between Cnet vs. F-V in walking and running ranged from: -0.59 and -0.64 for walking  and  -0.60 and -0.62 for running | None | 14 | P values ranged from 0.043 and 0.039 for the correlations between Cnet and F-L potential  P values ranged from 0.038 and 0.028 for the correlations between Cnet and F-V potential | None | Cnet vs. F-L potential  Cnet vs. F-V potential | Pearson’s correlation coefficient.  Pearson’s product moment correlation P values were corrected for multiple tests using the Benjamini–Hochberg procedure | None | Figure 4 and Table 2 | At any given speed, and for both tasks, the F-L and F-V potentials were negatively correlated with Cnet: the higher the force potentials the lower Cnet. |
